# ChIPXpress: using publicly available gene expression data to improve ChIP-seq and ChIP-chip target gene ranking

**DOI:** 10.1186/1471-2105-14-188

**Published:** 2013-06-10

**Authors:** George Wu, Hongkai Ji

**Affiliations:** 1 Department of Biostatistics, Johns Hopkins University Bloomberg School of Public Health, 615 North Wolfe Street, Baltimore, MD, 21205, USA

**Keywords:** ChIP-seq, ChIP-chip, Gene expression omnibus, Data integration, Gene regulation

## Abstract

**Background:**

ChIPx (i.e., ChIP-seq and ChIP-chip) is increasingly used to map genome-wide transcription factor (TF) binding sites. A single ChIPx experiment can identify thousands of TF bound genes, but typically only a fraction of these genes are functional targets that respond transcriptionally to perturbations of TF expression. To identify promising functional target genes for follow-up studies, researchers usually collect gene expression data from TF perturbation experiments to determine which of the TF targets respond transcriptionally to binding. Unfortunately, approximately 40% of ChIPx studies do not have accompanying gene expression data from TF perturbation experiments. For these studies, genes are often prioritized solely based on the binding strengths of ChIPx signals in order to choose follow-up candidates. ChIPXpress is a novel method that improves upon this ChIPx-only ranking approach by integrating ChIPx data with large amounts of **P**ublicly available gene **E**xpression **D**ata (PED).

**Results:**

We demonstrate that PED does contain useful information to identify functional TF target genes despite its inherent heterogeneity. A truncated absolute correlation measure is developed to better capture the regulatory relationships between TFs and their target genes in PED. By integrating the information from ChIPx and PED, ChIPXpress can significantly increase the chance of finding functional target genes responsive to TF perturbation among the top ranked genes. ChIPXpress is implemented as an easy-to-use R/Bioconductor package. We evaluate ChIPXpress using 10 different ChIPx datasets in mouse and human and find that ChIPXpress rankings are more accurate than rankings based solely on ChIPx data and may result in substantial improvement in prediction accuracy, irrespective of which peak calling algorithm is used to analyze the ChIPx data.

**Conclusions:**

ChIPXpress provides a new tool to better prioritize TF bound genes from ChIPx experiments for follow-up studies when investigators do not have their own gene expression data. It demonstrates that the regulatory information from PED can be used to boost ChIPx data analyses. It also represents an important step towards more fully utilizing the valuable, but highly heterogeneous data contained in public gene expression databases.

## Background

ChIPx, including ChIP-seq [1,2] and ChIP-chip [3,4], is a powerful technology for mapping transcription factor binding sites (TFBSs). Biologists often use it as a genome-wide screen to identify promising TF target genes to design follow-up studies or develop mechanistic hypotheses. A typical ChIPx experiment can identify thousands of TF bound genes. However, a large fraction of the bound genes are usually non-functional, in the sense that they do not respond transcriptionally to TF binding [5]. To help with identifying promising targets for functional studies, many researchers combine ChIPx data with gene expression data from TF perturbation experiments (i.e., experiments in which the TF is knocked down, knocked out, or over-expressed, etc.) to search for genes that are both bound by the TF and differentially expressed in the TF perturbation experiments. In this article, genes bound by the TF of interest are called TF “binding” targets, whereas genes that are both bound by the TF and transcriptionally respond to TF perturbation are defined as “functional” TF target genes. Since changes in TF binding at the functional targets are associated with changes in transcription, it is very likely that the binding changes at these genes will result in observable phenotype changes. For this reason, biologists often want to identify the functional targets among the binding targets to design follow-up studies because studying the functional genes is more likely to produce clearly interpretable results and may increase the success rate of the follow-up studies. Unfortunately, a large fraction of ChIPx studies do not have corresponding TF perturbation gene expression data. For instance, a survey of 58 published ChIPx studies randomly chosen from the Gene Expression Omnibus (GEO) [6] shows that around 40% (24/58) of the existing ChIPx studies do not have accompanying gene expression data. In these cases, investigators usually prioritize TF bound genes according to the strength of the ChIPx binding signals and then choose follow-up candidates from the top ranked genes, based on the assumption that the top ranked binding targets are more likely to be functional target genes than the lower ranked binding targets [7]. In order to improve upon this ChIPx-only based approach, we developed ChIPXpress to better identify functional TF target genes among TF binding targets when corresponding TF perturbation data is unavailable.

ChIPXpress is a novel method that relies on integrating large amounts of **P**ublicly available gene **E**xpression **D**ata (PED) with ChIPx data to better identify functional TF target genes. It is motivated by the observation that over 600,000 gene expression samples containing valuable biological information are currently deposited in the GEO, but remain relatively underutilized. The value of PED has been demonstrated by several groups for data mining in various applications such as tissue origin prediction and microarray sample phenotype identification [8-10]. However, using PED to improve other genomic analyses, such as ChIPx data analyses, particularly in complex genomes like human and mouse represents a novel research area that has yet to be extensively explored. Two complications that may arise when dealing with PED are its large heterogeneity, which may give rise to substantial lab and batch effects [11], and the overwhelming amount of data. Given its heterogeneity and size, much remains to be learned as to whether and how the wealth of information in PED can be effectively used to boost the analysis of other high-throughput genomic data. Moreover, the lack of tools to conveniently handle PED has also prevented many researchers from benefiting from these data in their daily research. Therefore, there is a need for easy-to-use but effective tools that can extract meaningful information from PED to enhance common genomic analyses. ChIPXpress helps to fill this role by providing a simple, fast and scalable algorithm that uses regulatory information from the diverse cell types, tissues, and diseases in PED to enhance functional TF target gene prediction from ChIPx data. By doing so, ChIPXpress also helps to increase the overall value of PED by providing a beneficial tool for researchers to more fully utilize the publicly available and useful gene expression data.

Ideally, researchers interested in using PED to improve functional target gene identification would extract only the relevant gene expression data in PED that applies to their ChIPx experiment. However, this approach has a number of limitations. First, it requires non-trivial and tedious manual work, which currently is a major barrier that discourages many researchers from using PED. For instance, researchers will need to collect data that matches the cell types and conditions of interest in their ChIPx experiment and then correctly analyze the data to identify differentially expressed genes. This requires careful reading of the database annotations to understand the sample origins and associated experimental design, as well as the biological and technical expertise to choose the most relevant samples and the most appropriate data analysis methods. If multiple TFs need to be analyzed and each TF has many matching gene expression datasets in PED, this process would need to be repeated multiple times, which may take a significant amount of effort and time. An alternative solution would be to automate the manual approach, but currently this is also very difficult and is not amenable to scale-up. The main issue with automation is it depends on the availability of high-quality and computer-friendly sample and experiment annotations, and requires effective tools for automatically extracting, accurately interpreting and analyzing the annotations. Maintaining high-quality annotations is non-trivial, as a lot of manual work needs to be invested during data storage, especially for a database that is currently growing at an exponential rate. On the other hand, tools for automatically parsing the annotations to accurately identify the samples, experiment design information, and the analysis methods are still far from mature. Thus, it remains difficult to automate this manual approach. Finally, this approach requires the availability of matching gene expression samples in PED. In many cases, especially if the investigator is the first to study the regulation of a set of TFs in a cell type of interest, corresponding gene expression data is simply unavailable since no one has yet to perform the relevant experiments.

Based on the reasons above, the goal of this article is to develop a method that can be easily used by researchers to explore the large and highly heterogeneous data in PED to extract useful biological information for identifying functional TF targets among the binding target genes detected in ChIPx experiments. We wish to develop an automated method that does not depend on sample and experiment annotations, so that it can be easily scaled up to accommodate the rapidly growing data in PED. Our solution, ChIPXpress, is constructed based on observing that the global correlation between a TF and its target genes across a large number of properly normalized PED samples from diverse cell types and biological conditions contains valuable information for identifying functional targets, in spite of the inherent heterogeneity of PED. We develop a simple ranking algorithm, based on a novel truncated absolute correlation measure, to combine ChIPx data and PED to more accurately predict functional TF target genes. The algorithm ranks genes predicted to be bound by the TF in ChIPx data by incorporating the TF binding information from ChIPx data and correlation in expression between TF and binding target genes from PED. The main purpose of ChIPXpress is to improve upon the TF-bound gene rankings generated from existing ChIPx peak calling tools to help identify the most promising functional target genes for follow-up studies. To evaluate this method, we applied it to 10 different mouse and human ChIPx data sets and found that ChIPXpress rankings consistently performed better than ChIPx-only rankings and may substantially increase the probability of finding functional TF targets in the top ranked genes, when researchers do not have their own TF perturbation gene expression data to accompany their ChIPx data. We also show that by using PED in its entirety, ChIPXpress makes it possible to improve functional target gene identification even when the gene expression data from the matching cell types and biological conditions are unavailable in public gene expression databases. ChIPXpress is implemented as an R/Bioconductor package which is freely available at the ChIPXpress website [12].

## Methods

### Data collection

We developed ChIPXpress using two large compendiums of human and mouse gene expression profiles collected from GEO. The human compendium consists of 18,257 gene expression samples from the Affymetrix Human U133 Plus 2.0 (GPL570) array, and the mouse compendium contains 9,634 samples from the Mouse 430 2.0 (GPL1261) array. Both these compendiums were compiled by McCall et al. [13,14]. Within each compendium, samples were consistently normalized using frozen RMA (fRMA) [13], and the normalized expression values of each gene across all samples were then standardized to have zero mean and unit standard deviation. For each gene with multiple probesets, only the probeset with the highest variance prior to standardization was retained.

fRMA is a new algorithm that can normalize tens of thousands of microarray samples collected by different labs from the same Affymetrix microarray platform. The algorithm normalizes all samples to a fixed (thus “frozen”) reference distribution. Since the reference distribution is frozen, one can process new array samples one-by-one without renormalizing the old samples. The normalized values from the new and old samples are directly comparable. In this way, one can easily expand the microarray compendium without incurring significant computational burden. fRMA can also more effectively reduce probe-related batch effects [13]. Using the fRMA normalized expression values, McCall et al. [14] have shown that within each microarray platform, the biological variation across the compendium samples from different cell types is larger than the lab or batch effects. As a result, the normalized expression values of the same gene can be meaningfully compared across samples in spite of their heterogeneous origins [14]. This is consistent with similar observations made by others [15].

**Figure 1:**
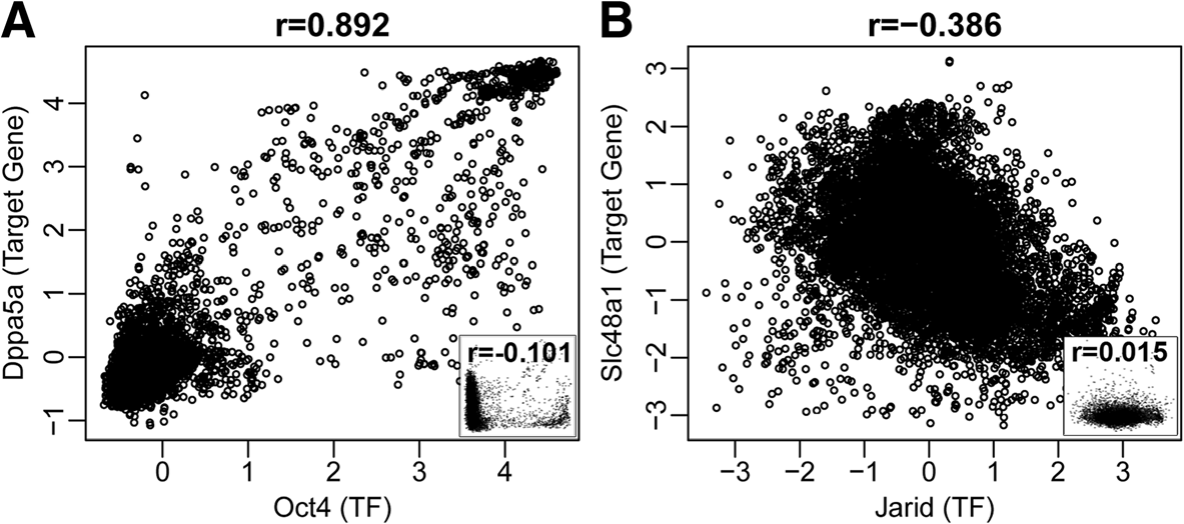
**Motivating examples of the value of PED.** (**A**) The TF *Oct4* shows strong correlation with its functional target gene *Dppa5a*, even though expression is measured from a diverse collection of tissues, diseases, and cell types by different labs. In contrast, a random gene only shows weak to zero correlation with the TF (bottom-right corner). (**B**) The transcriptional repressor *Jarid2* shows strong negative correlation with its functional target gene *Slc48a1*. Again, the correlation between the TF and a random gene is weak (bottom-right corner). The plots are based on 9,634 samples in a compendium of GPL1261 Affymetrix Mouse 430 2.0 arrays, where each dot corresponds to measurements from a single microarray sample.

Our exploration of these two PED compendiums further confirmed these observations, as we found that TFs and their known functional target genes (TG) are often highly correlated across compendium samples despite their diverse lab and cell type origins. For instance, the scatter plot in Figure 1A shows the fRMA normalized gene expression values for the TF *Oct4* and its known target *Dppa5a* across 9,634 mouse expression samples. The Pearson correlation coefficient between these two genes is strong, *r* = 0.892, especially given the heterogeneous lab and cell type origins of the compendium samples. In comparison, the correlation between *Oct4* and a randomly chosen gene is much weaker (*r* = −0.101) as shown in the bottom-right corner of the same plot. Using the Pearson correlation coefficients between *Oct4* and all genes as an empirical null distribution, the one-sided p-value for the observed *Oct4*-*Dppa5a* correlation (*r* = 0.892) is less than 0.001. Figure 1B provides another example where the expression of *Jarid2*, a known transcriptional repressor, and its repressed target gene *Slc48a1*, is shown across the same 9,634 mouse expression samples. Again, the Pearson correlation between these two genes is strong despite the heterogeneous lab and cell type origins of the compendium samples (*r* = −0.386, empirical p-value less than 0.003). In comparison, the correlation between *Jarid2* and a random gene is much weaker (*r* = 0.015) as shown in the bottom-right corner of the same plot. These examples suggest that the global correlation between a TF and a gene across the fRMA normalized PED samples from a diverse collection of cell types and conditions may correlate with the potential of a TF bound gene to become a functional target. This observation motivated the development of ChIPXpress which attempts to use the global TF-TG correlation across diverse PED samples to improve functional target gene prediction.

While the current implementation of ChIPXpress is based on the two pre-built PED compendiums described above, the ChIPXpress package also provides the users with tools to construct their own compendium of expression profiles from other Affymetrix platforms and species. The same processing procedure will be used to compile the new compendium; the gene expression data will be first consistently normalized and processed with fRMA and then standardized to have zero mean and unit standard deviation. Usage of these tools is described in full detail in the vignette manual of the R/Bioconductor package ChIPXpress.

### Truncated absolute correlation

**Figure 2:**
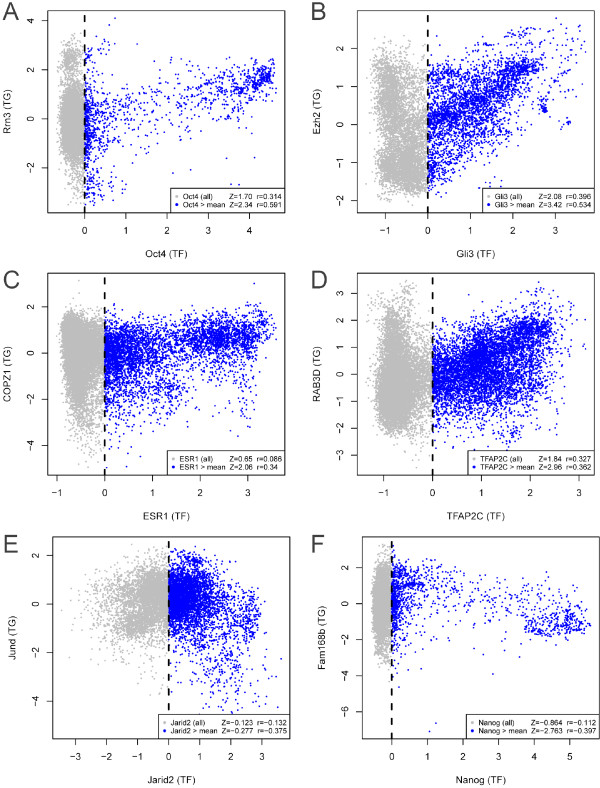
**Scatterplots of TF and target gene correlation and truncated correlation.** Plots of the expression of mouse and human transcription factors (TF) against the expression of one of their known target genes (TG): (**A**) *Oct4*, and the expression of its target gene, *Rrn3*, (**B**) *Gli3,* and the expression of its target gene, *Ezh2*, (**C**) *ESR1*, and the expression of its target gene, *COPZ1*, and (**D**) *TFAP2C,* and the expression of its target gene, *RAB3D,* (**E**) *Jarid2,* and the expression of its target gene, *Jund,* and (**F**) *Nanog,* and the expression of its target gene, *Fam168b*. In (**A,B,E,F**), each point is one of 9,634 samples in the GPL1261 mouse expression compendium and in (**C,D**), each point is one of 18,257 samples in the GPL570 human expression compendium. (**A-D**) *Oct4, Gli3, ESR1* and *TFAP2C* are examples of target gene activation by the TF, while (**E-F**) *Jarid2* and *Nanog* are examples of target gene repression by the TF. The Pearson correlation (*r*) between each TF and TG increases for activated target genes and decreases for repressed target genes when selecting only the subset of samples with higher than average TF expression (blue). Furthermore, if one constructs a null correlation distribution for each TF by calculating the correlation between each gene in the compendium with the TF, one can show that standardized Z-values obtained by subtracting the mean and dividing by the standard deviation of the null correlation increase in magnitude when excluding samples in which the TF expression is below the average TF expression. This shows that there is increased separation between the observed correlation and the null distribution.

One simple way to measure the global TF-TG correlation is the Pearson correlation coefficient, *r*. One may compute *r* between the TF-of-interest and each gene in the compendium to measure the regulatory potential. Genes with larger absolute values of *r* may have higher probability to be true functional targets. However, this simple correlation measure has a limitation; namely, it ignores the context-dependency of gene regulation. For example, we often observed that when the TF-of-interest is not expressed, its target genes could still be activated or repressed by other transcriptional regulators. Figure 2A shows the expression of *Oct4* and its known target *Rrn3* across all 9,634 mouse samples in the McCall compendium, where *Oct4* and *Rrn3* are positively correlated among samples with medium to high level of *Oct4* expression, but this correlation decreases substantially when *Oct4* expression is low. Among samples with low *Oct4* expression, many still have high *Rrn3* expression, possibly due to existence of other transcriptional regulators that can activate *Rrn3*. Thus, context-dependency of gene regulation may decrease the global correlation between TF and TG. When one uses all compendium samples to compute the Pearson correlation between *Oct4* and *Rrn3*, the correlation is *r* = 0.314. Using the Pearson correlation coefficients between *Oct4* and all genes to construct a null distribution, one can compute a standardized z-score for the observed *Oct4*-*Rrn3* correlation by subtracting the mean and dividing by the standard deviation of the null distribution. The resulting z-score is 1.70, which measures the separation between the observed correlation and the null. In contrast, when one excludes samples with low *Oct4* expression and only uses samples in which *Oct4* is above the average *Oct4* expression in the compendium to compute the Pearson correlation, the *Oct4*-*Rrn3* correlation increases to *r* = 0.591. One can use the same truncation approach to compute the correlation between *Oct4* and all other genes to obtain a null distribution and calculate a z-score. The z-score increases to 2.34, representing a better separation between the observed correlation and the null. Figure 2B-D shows a few additional examples for *Gli3* in mouse and *ESR1* and *TFAP2C* in human, where the correlation between TF and TG increases from 0.396 to 0.534, 0.086 to 0.34, and 0.327 to 0.362, respectively, when excluding samples in which the expression of the TF is below the average expression. Figure 2E-F shows an additional two examples for *Jarid2* and *Nanog* in mouse, in which the target gene expression is repressed by the TFs. In both cases, the negative correlation between the TF and TG became more negative when excluding the samples with below average TF expression. In all of these examples, the absolute value of the z-score increases irrespective of whether the TFs activate or repress their target gene. Since similar phenomenon was observed for many known TF-target gene pairs, we decided to use a new statistic, truncated absolute correlation, in ChIPXpress to more effectively measure the potential of a gene to be a functional TF target by taking into account the aforementioned context-dependency.

Assume there are *I* samples in the gene expression compendium. Let *x*_*ti*_ be the expression value of the TF-of-interest *t* in sample *i*, and *y*_*gi*_ be the expression of gene *g* in sample *i*. Given a cutoff value, *c*, let *C* = {*i*: *x*_*ti*_ ≥ *c*} denote the collection of samples in which the TF expression is bigger than or equal to *c*. The truncated Pearson correlation between the TF *t* and gene *g* is defined to be equal to the Pearson correlation coefficient calculated only using the samples in *C*:

$$r_{t,g}\;=\;\frac{\sum_{i\in C}\left(x_{\mathrm{ti}}\;-\;{\bar{x}}_{t}\;\right)\left(y_{\mathrm{gi}}\;-\;{\bar{y}}_{g}\right)}{\sqrt{\sum_{i\in C}{\left(x_{\mathrm{ti}}\;-\;{\bar{x}}_{t}\right)}^{2}\sum_{i\in C}{\left(y_{\mathrm{gi}}\;-\;{\bar{y}}_{g}\right)}^{2}}}$$

Here, $\bar{x}$ and $\bar{y}$ are averages taken across samples in *C*, and truncation refers to the exclusion of samples with low levels of TF expression (i.e., *x*_*ti*_ < *c*). This truncation can help with excluding samples in which a target gene is activated or repressed by other transcriptional regulators instead of the TF-of-interest, thus reducing the amount of noise by removing samples which are not likely to be informative for inferring the relationship between the TF-of-interest and its target genes. The truncated absolute correlation, *a*_*t,g*_ = | *r*_*t,g*_ | (i.e., the absolute value of *r*_*t,g*_), can then be used to measure the regulatory strength between the TF-of-interest and each gene in the compendium. Based on this definition, it is easy to see that the absolute value of the Pearson correlation coefficient is equivalent to a non-truncated absolute correlation, i.e. *a*_*t,g*_ where *c* = −∞. We will show later that the truncated absolute correlation performs better than the non-truncated absolute correlation.

### ChIPXpress ranking algorithm

Without gene expression data, one can prioritize TF bound genes based on the ChIPx signal alone. Using the PED compendiums, one can also rank the genes based on the truncated absolute correlation alone. ChIPXpress generalizes these two methods by combining ChIPx and PED information together in order to improve upon the ranking of TF bound genes identified from ChIPx experiments.

Suppose users have a list of TF bound genes ranked based on ChIPx data alone. The genes are ranked from the most to least likely to be regulated by a TF as determined by the ChIPx signal strength. Since most ChIPx peak calling algorithms rank their reported peaks, one can easily generate this list by ranking TF bound genes according to the highest-ranking peak associated with each gene. Let *P*_*g*_ (∈{1, …, *G*}) denote the ChIPx-only rank of gene *g*. For the TF-of-interest *t* and each TF bound gene *g*, one can also calculate the truncated absolute correlation *a*_*t,g*_ between *t* and *g* in the gene expression compendium using a set cutoff *c*. TF bound genes can then be ranked based on *a*_*t,g*_. Let *A*_*g*_ (∈{1, …, *G*}) be the rank of the TF bound gene *g* based on the truncated absolute correlation calculated from the gene expression compendium.

ChIPXpress combines the ChIPx-based rank *P*_*g*_ and the PED-based rank *A*_*g*_ through a linear combination *R*_*g*_ = w*P_*g*_ *+* (1*-w*)*A_*g*_ to produce a new ranking. If a TF bound gene does not have a corresponding expression measurement in the gene expression compendium, then the gene is not ranked by ChIPXpress. We note that, since the final ChIPXpress score *R*_*g*_ is a linear combination of ranks, a smaller *R*_*g*_ score will correspond to a higher ranked gene.

ChIPXpress has two parameters: the truncation cutoff *c* for computing the absolute correlation and the weight *w* for combining ChIPx and PED information. By default, ChIPXpress uses *c* = 0 and *w* = 0.1. This is based on our systematic tests below which show that irrespective of the (*c*, *w*) values, the rankings based on *R*_*g*_ are able to provide better or comparable overall performance compared to rankings based on ChIPx or PED data alone, and the optimal performance was achieved when *c* = 0 and *w* = 0.1. Note that since expression values of each gene are standardized to have zero mean, *c* = 0 amounts to using the samples in which the TF expression is above the average TF expression to compute the correlation.

### ChIPXpress implementation

ChIPXpress is currently offered as an R/Bioconductor package that can be used by any operating system that supports R. It can be downloaded and installed following the instructions at the ChIPXpress website [12]. Users will also need to download the accompanying ChIPXpressData package, which contains the pre-built mouse and human gene expression compendiums. If users would like to build their own gene expression compendium for a different platform or for a different species, the ChIPXpress package also provides the necessary functions to do so as explained in the software manual. To lower the loading time of the large amount of gene expression data, both compendiums are stored in big.matrix format using the bigmemory R package, which requires at least 64-bit of RAM for large databases (>2GB). Thus, users will be required to install the 64-bit version of R in order to have enough memory to load the compendiums.

## Results

### ChIPXpress is able to improve upon the ChIPx-only rankings

In order to evaluate ChIPXpress, we analyzed 10 ChIP-seq, ChIP-chip promoter array, and ChIP-chip whole-genome tiling array datasets for 9 different TFs – *Oct4, Jarid2, Gli3, Esrrb,* and *Nanog* in mice and *MYC, TFAP2C, HIF1A, and ESR1* in human (Table 1). We paired each dataset with a corresponding TF perturbation microarray or exon array dataset (i.e. gene expression profiles before and after alteration of TF expression) (Table 1). For each pair of ChIPx and TF perturbation data, we constructed gold standard functional TF target genes by intersecting the genes that were predicted to be bound by the TF in ChIPx data and differentially expressed in corresponding TF perturbation data. To determine TF bound genes, we processed ChIP-chip data using TileProbe [16] and the ChIP-seq data using CisGenome [17], and detected ChIPx peaks at the 10% false discovery rate (FDR) level. A gene is defined to be bound if there was at least one peak within 10 kb upstream and 5 kb downstream of the gene’s transcription start site. To determine the differentially expressed genes, we processed the TF perturbation data with RMA [18] for gene expression microarrays or GeneBASE implemented in JETTA [19] for exon microarrays to obtain normalized expression values. We then analyzed them with limma [20] to detect differentially expressed genes at a 10% FDR cutoff. Genes that are both bound in the ChIPx data and differentially expressed in the TF perturbation data are then labeled as gold standard functional targets. The gold standard target genes are listed in Additional file 1 and the number of gold standard target genes for each TF is listed in Table 1.

**Table 1:** Data used to evaluate ChIPXpress

**TF**	**Cell type**	**Species**	**Experiment type**	**Source**	**# of GS target genes**
Oct4	ESC	Mouse	ChIP-seq	GSE11724	
Oct4	ESC	Mouse	TFP - knockdown	GSE4189	
Oct4	ESC	Mouse			3010, 1303, 1224
Jarid2	ESC	Mouse	ChIP-chip (T)	GSE19167	
Jarid2	ESC	Mouse	TFP - knockout	GSE19165	
Jarid2	ESC	Mouse			1199, 370, 872
Gli3	Limbbud	Mouse	ChIP-chip (P)	GSE11062	
Gli3	Limbbud	Mouse	TFP - knockdown	GSE11062	
Gli3	Limbbud	Mouse			826, 712, 676
Esrrb	ESC	Mouse	ChIP-seq	GSE11431	
Esrrb	ESC	Mouse	TFP - knockdown	GSE13212	
Esrrb	ESC	Mouse			4826, 1591, 1377
Nanog	ESC	Mouse	ChIP-seq	GSE11431	
Nanog	ESC	Mouse	TFP - knockdown	GSE4189	
Nanog	ESC	Mouse			701, 560, 712
HIF1A	U87	Human	ChIP-chip (P)	GSE18499	
HIF1A	U251	Human	TFP - knockdown	GSE7835	
HIF1A	U251	Human			794, 1192, 725
ESR1	MCF7	Human	ChIP-chip (T)	GSE10800	
ESR1	MCF7	Human	TFP - overexpress	GSE11324	
ESR1	MCF7	Human			153, 564, 91
MYC	Helas3	Human	TFP - knockdown	GSE5823	
MYC	Helas3	Human	ChIP-seq	ENCODE-UTA	
MYC	Helas3	Human			768, 879, 1055
MYC	MCF7	Human	TFP - knockdown	GSE11791	
MYC	MCF7	Human	ChIP-seq	ENCODE-UTA	
MYC	MCF7	Human			836, 305, 901
TFAP2C	MCF7	Human	TFP - knockout	GSE8640	
TFAP2C	MCF7	Human	ChIP-seq	GSE21234	
TFAP2C	MCF7	Human			3289, 2302, 3504

*TFP* TF perturbation gene expression data, *P* Affymetrix Promoter array, *T* Affymetrix Tiling array, *GS* gold standard constructed by intersecting TF bound genes in ChIPx data with differentially expressed genes in TF perturbation experiments. For each TF, three sets of GS were constructed using three different peak callers. The table shows the number of GS target genes corresponding to TileProbe, MAT, TileMap for ChIP-chip data (left to right) and CisGenome, MACS, and Original for ChIP-seq data (left to right).

For each ChIPx dataset, we then pretended that the gene expression data from the TF perturbation experiments were not available, and used different methods to rank genes. The methods we tested include:

(1) ChIPXpress ranking, which ranks TF bound genes using the default parameter values *c* = 0 and *w* = 0.1.

(2) ChIPx-only ranking, which ranks TF bound genes by peak strength only using ChIPx data (i.e., peak ranks provided by the ChIPx peak caller).

(3) GEO-only ranking, which ranks genes by their truncated absolute correlation, obtained only using the PED from GEO.

If the TF perturbation experiments used to construct the gold standard genes were also contained in the PED compendium, the corresponding samples were removed from our PED compendium prior to the ChIPXpress analysis.

**Figure 3:**
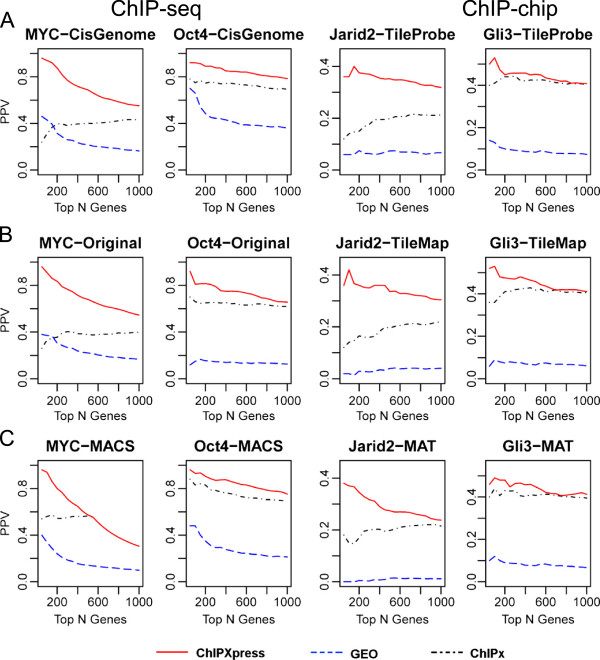
**PPV plot comparing prediction performance of ChIPXpress to ChIPx-only rankings and GEO-only rankings.** Prediction performance of ChIPXpress (solid) rankings compared to ChIPx-only (dot-dash) and GEO-only (dashed) rankings for *MYC* target genes in MCF7 cells*, Oct4* target genes in embryonic stem cells (ESCs), *Jarid2* target genes in ESCs, and *Gli3* target genes in limbbud. The positive predictive value (PPV) is depicted for the top 50, 100, …, 1000 predictions. Three different peak technologies are evaluated for each TF: (left-right) ChIP-seq, (column 1–2), ChIP-chip whole genome tiling arrays (column 3), and ChIP-chip promoter arrays (column 4). Three different sets of peak callers are also tested for each TF: (**A**) CisGenome for ChIP-seq, and TileProbe for ChIP-chip, (**B**) Original (the peaks reported by the original publication) for ChIP-seq, and TileMap for ChIP-chip, and (**C**) MACS for ChIP-seq, and MAT for ChIP-chip.

For each test dataset, we compared different ranking methods based on their ability to identify gold standard functional TF target genes among the top ranked genes. In this regard, we computed the percentage of top ranked targets that were true functional targets, or the positive predictive value (PPV), for each method. The PPVs of different methods were then compared. The results from the ten test datasets were largely consistent, and Figure 3A shows four representative examples. For each dataset, the figure shows three curves, corresponding to the PPVs for ChIPXpress, ChIPx-only and GEO-only rankings, respectively, versus increasing predicted target gene list size. ChIPXpress outperformed both the ChIPx-only ranking and the GEO-only ranking, and provided the highest percentage of true functional target genes among the top ranked genes. The level of improvement varied across datasets and was substantial in some cases (e.g. *MYC* and *Jarid2*). These analyses show that incorporating PED can improve upon the ChIPx-only ranking when an investigator does not have his/her own TF perturbation gene expression data.

### Prediction improvement is not tied to a specific ChIPx platform or peak calling algorithm

Next, we asked whether the observed improvement brought by ChIPXpress was tied to a specific ChIPx peak calling algorithm. To answer this question, we re-defined TF bound genes with several other peak calling algorithms by detecting ChIPx peaks using their default parameter settings, and reevaluated ChIPXpress prediction performance. These additional algorithms include MAT [21] and TileMap [22] for ChIP-chip data, and MACS [7] and Original (i.e., the peak ranking reported by the original paper that generated the ChIPx data) for ChIP-seq data. For each test dataset and peak calling algorithm, we evaluated the ChIPXpress, ChIPx-only and GEO-only rankings. In each evaluation, the same peak calling algorithm and parameters were used to obtain the ChIPXpress rankings, the ChIPx-only rankings, and the TF bound genes used to construct the gold standard. We found that ChIPXpress was able to improve upon the ChIPx-only ranking irrespective of which ChIPx peak caller was used. For instance, Figure 3B,C shows the results for the same four datasets in Figure 3A. In all cases, ChIPXpress provided the best ranking performance. The four datasets in Figure 3 represented three different platforms for ChIPx experiments (ChIP-seq, ChIP-chip promoter arrays, and ChIP-chip whole genome tiling arrays). Altogether, our results suggest that ChIPXpress is able to improve functional target gene identification irrespective of the ChIPx platform and peak caller.

### ChIPXpress robustly improves ranking for a wide range of parameter values

The comparisons so far were based on the default parameters of ChIPXpress. We also studied the performance of ChIPXpress using a wide range of other parameter values. To facilitate the comparison, we summarized the ranking performance of each method in each dataset into a normalized AUC score, calculated using the Area Under the PPV Curve divided by the total plot area for the top *n* (*=*100, 500 and 1000) predictions. For instance, for each curve in Figure 3 and each *n* in {100, 500, 1000}, this amounts to computing the average of the positive prediction values across the top *n* predictions (i.e., adding the positive predictive value at each possible prediction list size from 1 to *n* and then dividing by *n*). Since 10 datasets were analyzed, 10 normalized AUC (nAUC) scores were obtained for each *n* and each ranking method (ChIPx-only, GEO-only, or ChIPXpress using different parameter values).

**Figure 4:**
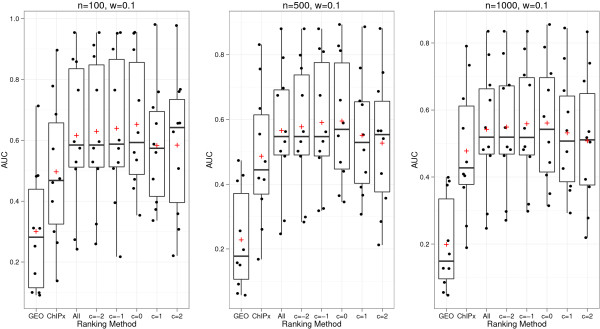
**Evaluation of ChIPXpress performance across different truncation cutoffs for a set ranking weight of 0.1.** Boxplot of the performance of different ranking methods for GEO-only, ChIPx-only, and ChIPXpress using TF expression cutoffs of *c* = −∞ (All), -2, -1, 0, 1, 2. The ranking weight is set to *w* = 0.1 for each method tested. Performance is measured by the normalized AUC score (the area under the positive predictive value curve divided by the total plot area). This is equivalent to averaging the positive predictive values across the top 1 to *n* (100, 500, 1000) ranked predictions. Each individual point corresponds to a specific TF data set and the crosses correspond to the mean normalized AUC score across all datasets tested. CisGenome was used to analyze ChIP-seq data and TileProbe was used to analyze ChIP-chip data.

We first fixed the weight parameter *w* to its default value 0.1 and asked how changing the truncation cutoff *c* may change the nAUC scores. Six different cutoff values were tested: *c* = −∞, -2, -1, 0, 1 and 2. Figure 4 shows the results when the ChIP-seq and ChIP-chip peaks were called using CisGenome and TileProbe respectively. For each ranking method, including ChIPx-only, GEO-only, and ChIPXpress using different values of *c*, the distribution of the 10 nAUC scores is shown as a box plot. In the figure, “All” corresponds to ChIPXpress with *c* = −∞, which is equivalent to computing the traditional Pearson correlation coefficients using all the samples in the compendium and then using their absolute values to rank genes. Comparing the nAUC scores of different ranking methods, one can see that regardless of which parameter values were used for the truncation cutoff *c*, ChIPXpress robustly performed better than or comparable to the ChIPx-only and GEO-only rankings. The figure also shows that *c* = 0 produced the best overall performance among all the tested methods. This can be seen by tracking the average nAUC score across different values of *c*. Comparing the results for *c* = −∞ and the results for *c* = 0, one can also see that using the truncated absolute correlation can improve ranking compared to using the non-truncated absolute correlation (e.g., the mean and minimal nAUC scores for *c* = 0 were bigger than the mean and minimal nAUC scores for *c* = −∞).

**Figure 5:**
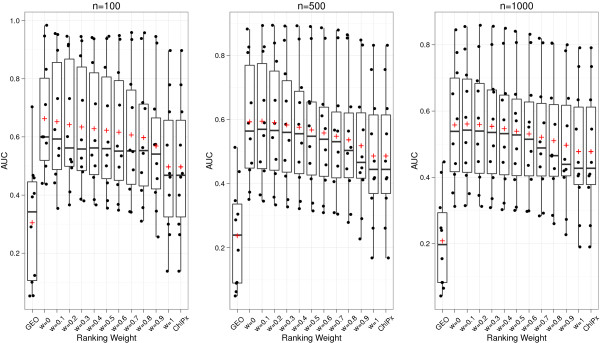
**Evaluation of ChIPXpress performance across different rankings weight for a set truncation cutoff of 0.** Boxplot of the performance of different ranking methods for GEO-only, ChIPx-only, and ChIPXpress using weights of *w* = 0, 0.1, 0.2, … , 1. The TF expression cutoff is set to *c* = 0 for each weight tested. Performance is measured by the normalized AUC score (the area under the positive predictive value curve divided by the total plot area). This is equivalent to averaging the positive predictive values across the top 1 to *n* (100, 500, 1000) ranked predictions. Each individual point corresponds to a specific TF data set and the crosses correspond to the mean normalized AUC score across all datasets tested. CisGenome was used to analyze ChIP-seq data and TileProbe was used to analyze ChIP-chip data.

Next, we fixed the truncation cutoff *c* to its default value 0 and asked how changing the weight parameter *w* may change the nAUC scores. Eleven different *w* values were tested: *w* = 0, 0.1, 0.2, …, 1. Figure 5 shows the nAUC scores of ChIPXpress obtained using different *w* values. Here, peaks were also called using CisGenome and TileProbe. Based on the figure, *w* = 0.1 produced the best overall performance among all the tested methods. *w* = 0.1 had the highest mean and median nAUC for *n* = 500, 1000, and *w* = 0 had the highest mean and median nAUC for *n* = 100. Similar to before, we found that ChIPXpress rankings were better (or no worse) than both the GEO-only rankings and the ChIPx-only rankings irrespective of which weight *w* was used to combine the ChIPx and PED information. It is important to note that when *w* = 1, the ChIPXpress ranking is the same as the ChIPx-only ranking. However, when *w* = 0, the ChIPXpress ranking is not the same as the GEO-only ranking (Figure 5). This is because in the GEO-only ranking, no information from ChIPx data, namely whether a gene is TF bound or not, is incorporated, thus all genes in the compendium are ranked by the absolute correlation *a*_*t,g*_. In contrast, the ChIPXpress ranking with *w* = 0 first extracts the subset of genes that are TF bound, and then ranks the TF bound genes by *a*_*t,g*_. Therefore, ChIPx information is still used for determining which genes are TF bound. This means that even though the default weight *w* = 0.1 for the ChIPx data seems to be small, the actual contribution of the ChIPx data to the final gene ranking is more than the 10% represented by the weight parameter *w*.

**Figure 6:**
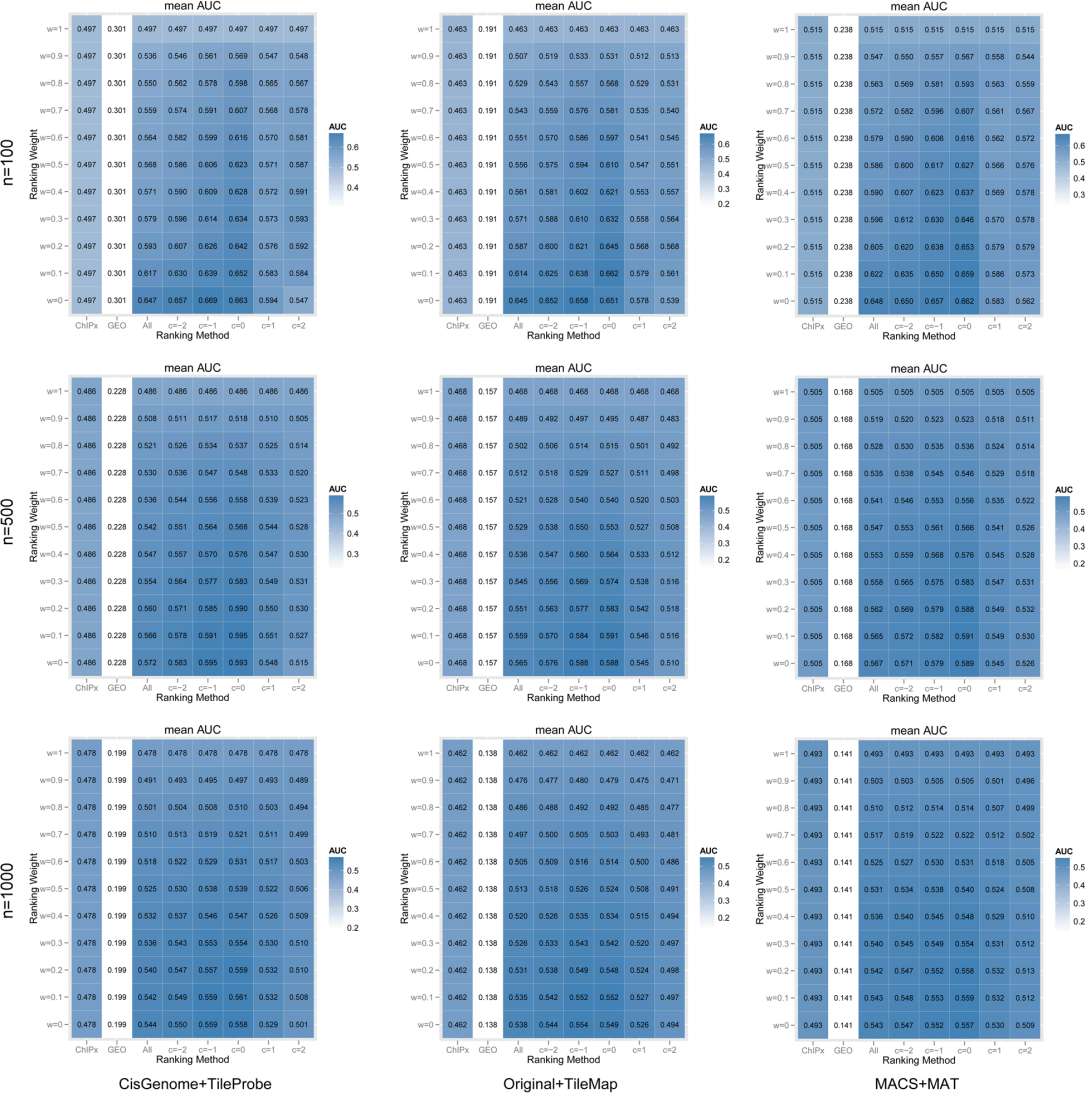
**Evaluation of ChIPXpress ranking performance across different parameter combinations.** Heat map of average normalized AUC scores for different combinations of ranking methods and ranking parameters across all datasets tested using CisGenome and TileProbe (left), Original and TileMap (middle), and MACS and MAT (right) to analyze the test ChIPx datasets. The ranking methods evaluated include GEO-only, ChIPx-only, and ChIPXpress using a combination of cutoff values of *c* = −∞ (All), -2, -1, 0, 1, 2, with ranking weights of *w* = 0, 0.1, 0.2, … , 1. Performance is measured by averaging the normalized AUC score (the area under the positive predictive value curve divided by the total plot area) across all tested datasets. Overall, the best performing ranking method and weight is ChIPXpress with *c* = 0 and *w* = 0.1.

Figures 4 and 5 only show the prediction performance across the 10 test ChIPx datasets marginally (e.g. either *w* or *c* must be fixed, while the other parameter is allowed to vary). To visualize the prediction performance when both *w* and *c* are jointly allowed to vary, we constructed heat maps showing the average nAUC score of the 10 tested datasets for each *w* and *c* pair (Figure 6). Figure 6 also shows the results obtained using other peak calling algorithms to determine TF bound genes. Since the 10 test datasets contained both ChIP-chip and ChIP-seq data, we paired the possible peak callers into 3 different ChIPx analysis methods: CisGenome (ChIP-seq) with TileProbe (ChIP-chip), MACS (ChIP-seq) with MAT (ChIP-chip), and Original (ChIP-seq) with TileMap (ChIP-chip). Figure 6 shows that overall, ChIPXpress performed better than both the GEO-only ranking and the ChIPx-only ranking irrespective of which ChIPx analysis method and parameter values (*w*, *c*) were used. When *w* = 0.1 and *c* = 0, ChIPXpress achieved its best overall prediction performance. Specifically, out of the 9 possible combinations between *n* = 100, 500, 1000 and the 3 ChIPx analysis methods, *w* = 0.1 and *c* = 0 offered the highest (or tied for the highest) mean nAUC among the tested (*w, c*) parameter values for 6 out of the 9 combinations. Based on these results, we set the ChIPXpress default procedure to first rank the predicted TF-bound genes by calculating *a*_*t,g*_ by using only the samples in which the TF expression is above the average TF expression (*c* = 0) and then calculate a final ChIPXpress ranking by combining the ChIPx ranking and *a*_*t,g*_ ranking with weights 0.1 and 0.9, respectively (*w* = 0.1).

### ChIPXpress is scalable, efficient, and fast

Since ChIPXpress does not depend on sample annotations and is automated, it can be easily applied in a high-throughput fashion. For instance, ChIPXpress was recently adopted by hmChIP [23], a database for publicly available ChIP-seq and ChIP-chip data in human and mouse, to provide improved target gene rankings for 400+ TF ChIPx datasets. These data can be downloaded from the hmChIP website [24]. Processing these 400+ datasets using ChIPXpress only took 1 hour on an 800 MHz Quad-core AMD Opteron™ Processor 2384 computer with 4GB of memory. A similar manual analysis of the 400+ TF ChIPx datasets would be more difficult and much more time consuming. Thus, ChIPXpress can dramatically increase efficiency, which is essential for scale-up.

### ChIPXpress may be applied when no matching expression data are available in PED

Our analyses above show that the global TF-TG correlation, *a*_*t,g*_, which reflects the potential of a gene to co-express (correlate or anti-correlate) with the TF, provides useful information to help identify a truly functional TF target gene. This global correlation is supported by large amount of data from a diverse collection of cell types and biological conditions. Therefore, removing one or two cell types from the PED compendium usually has little effect on *a*_*t,g*_. In this sense, the global TF-TG correlation is a general characteristic of a gene that is not contingent on the availability of one specific cell type in PED. In ChIPXpress, this global expression correlation is combined with the cell type and condition dependent binding information in the ChIPx data to improve target gene prediction. Genes with large expression correlation but no binding in the experimental condition of interest or genes with strong binding but weak expression correlation will not be picked up by ChIPXpress.

**Figure 7:**
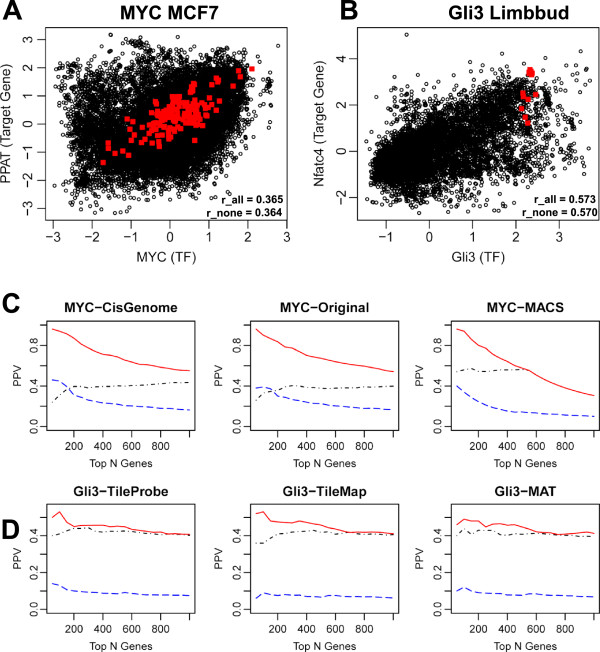
**PPV plot of ChIPXpress prediction performance when no matching gene expression data in PED is available.** Plot of MYC expression compared to an example target gene, *PPAT* (**A**), and *Gli3* expression compared to an example target gene, *Nfatc4* (**B**), demonstrating similar levels of TF-TG correlation, even after removing all MCF7 and limbbud related cell types in the human and mouse PED compendium, respectively. Prediction performance of ChIPXpress (solid) rankings is compared to ChIPx-only (dot-dash) and GEO-only (dashed) rankings for *MYC* target genes in MCF7 cells (**C**)*,* and *Gli3* target genes in limbbud (**D**). The positive predictive value (PPV) is depicted for the top 50, 100, …, 1000 predictions In both analyses, all related cell types are removed from the compendium to simulate situations in which gene expression data related to the cell-type of interest is unavailable.

Since adding or removing a cell type or condition from the PED compendium usually has little effect on the global TF-TG correlation, we speculate that it might be possible to apply ChIPXpress even when no gene expression data matching the cell type and condition used for the ChIPx experiment are available in PED. To test whether this is the case, we analyzed two ChIPx datasets in Table 1 – human *MYC* in MCF7 cells and mouse *Gli3* in limb bud – but only after removing all related samples to the experimental cell type or tissue (i.e., MCF7 and limb bud) in the gene expression compendium. Removing these samples was done by manually examining cell type annotations of all samples in the compendium. Since this is a time-consuming process, we only analyzed the two ChIPx datasets above. Figure 7A-B shows the PED samples removed (red) and retained (black) for the ChIPXpress analysis in two examples. This figure shows that the removed samples are only a small fraction of all samples, and the global TF-TG correlation pattern remains clear even when the red dots are not considered. In Figure 7C-D, we performed analyses similar to Figure 3 after removing the MCF7 and limb bud samples. Comparing Figure 7C-D and Figure 3, one can see that removing the samples with matching cell types from PED had little to no effect on ChIPXpress prediction performance. Most importantly, ChIPXpress rankings still performed better than both the ChIPx-only and the GEO-only rankings. These examples demonstrate that even when no gene expression experiments have been performed in the cell type or condition corresponding to the ChIPx experiment, it is still possible to use ChIPXpress to improve functional target gene ranking compared to using only the ChIPx data.

We also note that while the global TF-TG correlation computed using PED does not depend on a specific cell type or condition, it is combined with cell type and condition dependent binding information to produce the final ranking, and therefore the ChIPXpress ranking is still cell type and condition dependent (e.g., genes with large “potential” to globally co-express with the TF but no binding in the experimental condition in question will not be ranked by ChIPXpress). To demonstrate, the top 100, 500, and 1000 genes identified by ChIPXpress using CisGenome from the two MYC datasets in Table 1 – one from MCF7 and one from HelaS3 cells - had 32.0%, 27.2%, and 22.8% overlap, respectively. Thus, the large majority of highly ranked MYC target genes in HelaS3 and MCF7 cells are still cell-type specific, even though the rankings utilize a common measure of TF-TG correlation in PED.

### ChIPXpress sample commands

ChIPXpress is implemented as an R/Bioconductor package which can be easily used by researchers. As an example, below we provide the commands to reproduce the *Oct4* ChIPXpress analyses described in the paper. Users will first need to download and install the ChIPXpress and ChIPXpress data packages. The ChIPXpress package contains the necessary ranking functions and the ChIPXpress data package contains the pre-built mouse gene expression compendium (GPL1261) used in the paper.

Next, users will need to load the packages and the mouse gene expression compendium.

library(ChIPXpress)

library(ChIPXpressData)

library(bigmemory)

path < − system.file(“extdata”, package = “ChIPXpress”)

DB < − attach.big.matrix(“DB_GPL1261.bigmemory.desc”,path)

Then they will need to specify the Entrez Gene ID for the TF-of-interest, *Oct4*, and a ranked list of predicted TF bound genes from ChIPx data.

TFID < − “18999”

data(Oct4ESC_ChIPgenes)

Finally, users will input the above information into the ChIPXpress ranking function:

ChIPXpress(TFID,Oct4_ChIPgenes,DB)

The function will output two results. The first is a ranked vector of *Oct4* bound genes sorted from the smallest to largest *R*_*g*_. The second contains the remaining *Oct4* bound genes that were not found in the compendium. Full details and more examples, including how users can build their own compendium for different platform or species, can be found in the accompanying vignette and help files in the R/Bioconductor package ChIPXpress.

## Discussion

We have demonstrated that by using a simple algorithm, ChIPXpress is able to produce more accurate functional TF target gene rankings than ChIPx-only and GEO-only rankings. The method is amenable to scale-up as it does not require sample annotations. The R/Bioconductor package makes ChIPXpress easy-to-use for individual investigators. In addition, our analyses show that it is possible to use ChIPXpress to improve target gene ranking even when no related gene expression data is available in PED. ChIPXpress can help to alleviate many drawbacks that affect manual approaches to identify functional target genes from ChIPx data. Therefore it provides a valuable tool for TF target gene prioritization when users want to analyze their own ChIPx data but do not have accompanying gene expression data from TF perturbation experiments. Since most ChIPx studies and peak detection algorithms provide a list of TF-bound genes ranked by binding strength, investigators can also easily use ChIPXpress to quickly survey other ChIPx experiments relevant to the TF and cell type of their interest.

Although our results show that ChIPXpress is able to improve functional target gene identification by combining publicly available gene expression data and ChIPx data, it is important to mention a few caveats.

First, our truncated absolute correlation measure will not be able to properly rank genes and transcription factors that are not measured reliably by microarrays. This will be the case especially for lowly expressed genes or transcription factors that are regulated primarily through post-translational modifications. Fortunately, based on our experience this does not affect the large majority of genes and transcription factors, but users will still need to be aware of this issue when analyzing their own ChIPx data in case this issue affects their TF-of-interest. In cases when the TF-of-interest is consistently expressed at very low levels or shows little variation in expression across all samples in the PED compendium, the TF-TG expression correlation may not provide as much useful information. To alert users of this possibility, ChIPXpress reports the mean, variance, and coefficient of variation for the queried TF computed using expression values before standardization. Then users will be warned if any of the three statistics fall below the 5th or 25th quantile of the distribution of the mean, variance, and coefficient of variation for all genes in the compendium.

Second, our target gene rankings assume that functional TF binding will induce significant changes in the expression of its target genes. This is not always true. For example, housekeeping genes can be expressed at a stable level across a variety of cell types and conditions. This may be because many housekeeping genes are activated by multiple TFs in different cell types to ensure consistent expression of the genes. As a result, binding changes in one TF in a specific cell type may not induce changes in the expression of these genes. If users are more interested in all TF target genes irrelevant of whether or not the target gene is transcriptionally affected by TF binding, then ChIPXpress will not be useful. However, since a large number of investigators – especially, in initial functional studies to determine which target genes of a TF should be selected for follow-up - are interested in target genes that exhibit substantial transcriptional response to TF binding, ChIPXpress is still broadly applicable.

Third, our modified Pearson correlation coefficient is not necessarily the best and only way to capture the TF-TG regulatory information contained in PED. Although we have shown that it does improve upon the naive Pearson correlation coefficient by focusing on the samples in which the TF is likely to be expressed, other measures that are more robust or accurate may exist and are worthwhile to explore in the future. For instance, the global TF-TG correlation used to characterize the possibility of a gene to become a functional target is based on large amounts of data, and a strong correlation is often supported by multiple cell types and conditions. If a TF target gene is functional in only one cell type or condition and the gene does not respond to the TF binding in all other cell types or conditions, then it is possible that the global correlation between the TF and TG across all PED samples is very weak. In such a scenario, when ChIPx data is collected from the cell type/condition in which the target gene is functional, ChIPXpress may not rank that gene among the top due to its weak global correlation with the TF. How to better identify these types of genes is still under investigation.

Despite these limitations, it is important to remember that only after having established the feasibility and concept that the huge and heterogeneous PED can be used to improve ChIPx data analysis, are we then able to pursue the other avenues of improvement through more sophisticated models and measures. One major contribution of the current work is establishing the feasibility of our approach in complex vertebrate genomes.

In this article, the gold standard target genes used to evaluate ChIPXpress were obtained by combining experimental ChIPx data with TF perturbation gene expression data. A method frequently used to assess ChIPx peak callers [25] is to measure the motif occurrence rate among the top ranked peaks. This method was not used here for evaluation because our goal is to improve the prediction of functional target genes among the TF-bound genes rather than detecting the TF binding targets *per se*. Previous studies have shown that a significant fraction of TF binding targets with motifs and reliable ChIP-seq signals are not functional [5,26]. Thus, while the motif occurrence rate is a natural measure to evaluate quality of the binding peak calls, i.e. whether a genomic locus is bound or not by the TF, it does not directly provide information needed for evaluating the functionality of the target genes, i.e., whether the TF binding at a gene will result in transcriptional response. Functional evidence would require a change in the expression of the gene in response to binding, which can only be determined from additional TF perturbation gene expression data rather than the presence or absence of TF motifs. This is the reason why TF perturbation experiments are often performed in addition to ChIPx experiments. It also explains why we relied on TF perturbation gene expression data rather than motif occurrence rate to evaluate the effectiveness of the ChIPXpress algorithm. Despite this potential limitation of motif-based evaluation, we did perform a comparison between ChIPXpress, ChIPx-only and GEO-only rankings based on motif occurrence rates. This comparison is presented in Additional file 2 for the reader’s reference. The analysis shows that for the majority of the data analyzed here, ChIPXpress rankings are associated with comparable or better motif occurrence rates compared to the other ranking methods.

Currently, ChIPXpress is designed to use large compendiums of gene expression microarray data, but the same approach in principle can also be used with RNA-Seq data [27]. This might improve the performance of ChIPXpress since RNA-seq is known to be able to provide more accurate gene expression estimates than gene expression microarrays. Although this is true, we first focused on gene expression microarray data because there are over 600,000+ gene expression microarray samples compared to 10,000+ RNA-seq samples currently deposited in GEO. The significantly larger number of microarray samples provide a much wider variety of cell types, tissues, and diseases that allows the truncated absolute correlation measure to more accurately capture the regulatory potential between each gene and TF. Moreover, building a large compendium of RNA-seq samples is not an easy or straightforward task. Publicly available RNA-seq data is generated from many different platforms and processed using a variety of algorithms. Current understanding of how to properly normalize the data to combine them together and analyze them jointly is very limited. Consistent methods to handle large RNA-seq datasets still need to be developed and explored to even begin utilizing the data to improve ChIPx analyses or other functional analyses. Rather than waiting for RNA-seq methodology to mature and more RNA-seq data to be deposited in PED, ChIPXpress can be used immediately to improve ChIPx analyses by taking advantage of the large amounts of gene expression microarray data in PED currently available. This explains why the current implementation of ChIPXpress is based on gene expression microarray data. In the future, as the RNA-seq data continue to grow and the methods for analyzing those data become mature, it should be more straight-forward to extend ChIPXpress to also accommodate RNA-seq data.

## Conclusions

In summary, ChIPXpress improves functional target gene identification from ChIPx data using publicly available gene expression data in a simple and straightforward manner. Our results show that when an investigator does not have his/her own TF perturbation data, ChIPXpress is able to more accurately rank functional TF target genes compared to rankings obtained using only ChIPx data, which may significantly increase the chances of finding real functional TF targets among the top ranked genes. ChIPXpress demonstrates the value of PED and hopefully encourages more research focused on improving ChIPx data analysis by incorporating PED.

## Competing interests

The authors declare that they have no competing interests.

## Authors’ contributions

GW designed and implemented the ChIPXpress method and performed the analyses. HJ designed and supervised the development of ChIPXpress. Both authors wrote and drafted the manuscript. All authors read and approved the final manuscript.

## Supplementary Material

Additional file 1 **Gold standard target genes as listed in Table **1**.** Click here for file

Additional file 2 Evaluation of motif occurrence rate comparing ChIPXpress rankings to ChIPx-only and GEO-only rankings. Click here for file
