# Supplementary material for: ChIPXpress: using publicly available gene expression data to improve ChIP-seq and ChIP-chip target gene ranking
Source: BMC Bioinformatics. 2013 Jun 10;14:188. doi: 10.1186/1471-2105-14-188 (PMC3684512; doi:10.1186/1471-2105-14-188)
Supplement: Additional file 2 — Evaluation of motif occurrence rate comparing ChIPXpress rankings to ChIPx-only and GEO-only rankings. [file 1471-2105-14-188-S2.pdf]

Additional file 2

Evaluation of ChIPXpress ranking using motif occurrence rates

In the main article, we demonstrated that ChIPXpress was better able to rank functional target genes by evaluating how many experimentally defined gold standard functional targets were recovered among the top ranked genes. An alternative approach that is commonly used in the ChIPx community to compare the performance of ChIPx peak detection algorithms is to calculate how often the transcription factor binding motif occurs in the detected peaks, where higher motif occurrence rates would correspond to more accurate peak detection [1-2]. The advantage of this approach is that DNA motif information may be able to provide an independent evaluation of prediction performance. Unfortunately, although motif occurrence rate can be used to measure binding target prediction accuracy, it is unable to directly measure functional target gene prediction accuracy. The main problem is that the presence of a motif in the binding sites associated with a gene is not sufficient to identify whether the gene is a functional target of a TF. Past research has been shown that a large fraction of binding sites predicted from ChIPx data, even with the presence of the TF motif, are non-functional and do not play a meaningful biological role [3-4]. This is the primary reason why most researchers perform additional gene expression experiments, in which the expression of the TF is perturbed, along with the ChIPx experiments to determine which genes actually respond transcriptionally to TF binding. Thus, evaluation of motif occurrence rates is not guaranteed to be an appropriate measure of functional target prediction accuracy, and at most, it can only serve as an approximate indirect measure of functional target gene prediction accuracy. This explains why we mainly relied on the TF perturbation gene expression data to construct gold standard for evaluating ChIPXpress, where the goal is to identify functional target genes among binding targets.

Despite the limitations of motif-based evaluation, we also did a comparison of ChIPXpress, ChIPx-only and GEO-only gene rankings using motif occurrence rates for the reader’s reference. Since using different peak calling algorithms to derive ChIPXpress and ChIPx-only gene rankings produced

similar motif comparison results, below we illustrate the main findings using the results based on CisGenome (for ChIP-seq peak calling) and TileProbe (for ChIP-chip peak calling). First, motifs for each of the nine TFs tested were found by searching through the TRANSFAC [5] and JASPAR [6] motif databases. We were able to identify a corresponding motif for each TF, except for *Jarid2* (**Supplementary Table 1**). Then using the 9 datasets corresponding to the 8 recovered TF motifs (**Table 1**), we calculated the motif occurrence rate among the top ranking genes for each of the three ranking methods: ChIPXpress, ChIPx-only, and GEO-only. To calculate motif occurrence rates, we first extracted the 500 base pair region surrounding the center of each TF binding peak (i.e., the center of each predicted peak was extended on both sides by 250 base pairs). We then mapped motifs to these regions using CisGenome with its default parameter settings. Based on the mapping results, motif occurrence for a given gene was defined as the absence or presence of a motif in any of the peaks assigned to a gene, where peaks were assigned to a gene if they overlapped with the +10 kbp upstream to -5 kbp downstream region around the gene's TSS. Then the average motif occurrence for the top *N* genes was defined as the motif occurrence rate. In other words, if a gene has a peak that contains at least one motif site, then the gene is counted as having a motif-containing peak and the percentage of genes with at least one motif-containing peak is the motif occurrence rate.

We found that across the 9 datasets, ChIPXpress improved motif occurrence rates compared to ChIPx-only rankings for 4 of the 9 datasets: *HIF1A* in U87 cells, *MYC* in HelaS3 cells, *MYC* in MCF7 cells, and *Nanog* in ESCs (**Supplementary Figure 1**). Similar motif occurrence rates were found in 4 of the 9 datasets: *ESR1* in MCF7 cells, *Oct4* in ESCs, *TFAP2C* in MCF7 cells, and *Esrrb* in ESCs. Only for 1 of the 9 datasets - *Gli3* in limbbud - was there a clear overall decrease in motif occurrence rate. We note that for the *Gli3* data, ChIPXpress performed better than ChIPx-only in **Figure 3**. Possible reasons could be the functional targets in limbbud have a slightly different motif than the motif in TRANSFAC, or cell-type specific collaborating factors not studied here may be influencing the functional consequences of *Gli3* binding in limbbud. Thus, a motif-containing peak does not necessarily imply the binding is

functional (therefore the higher motif occurrence rate associated with ChIPx-only ranking does not translate into a higher functional target gene prediction accuracy determined by TF perturbation data). GEO-only rankings, on the other hand, performed significantly worse for all 9 data sets. Overall, our analysis shows that in the majority of the test datasets (8/9 for the ChIPXpress vs. ChIPx-only comparisons, and 9/9 for the ChIPXpress vs. GEO-only comparisons), ChIPXpress provided comparable or better motif occurrence rates.

Supplementary Table 1 – List of TF Motifs

Motif	Species	Database	ID
HIF1A	Human	TRANSFAC	M00466
MYC	Human	TRANSFAC	M00799
ESR1	Human	JASPAR	MA0112.2
TFAP2C	Human	TRANSFAC	M00470
Nanog	Mouse	TRANSFAC	M01123
Oct4	Mouse	TRANSFAC	M01125
Esrrb	Mouse	JASPAR	MA0141.1
Gli3	Mouse	TRANSFAC	M01037
Jarid2	Mouse	-	-

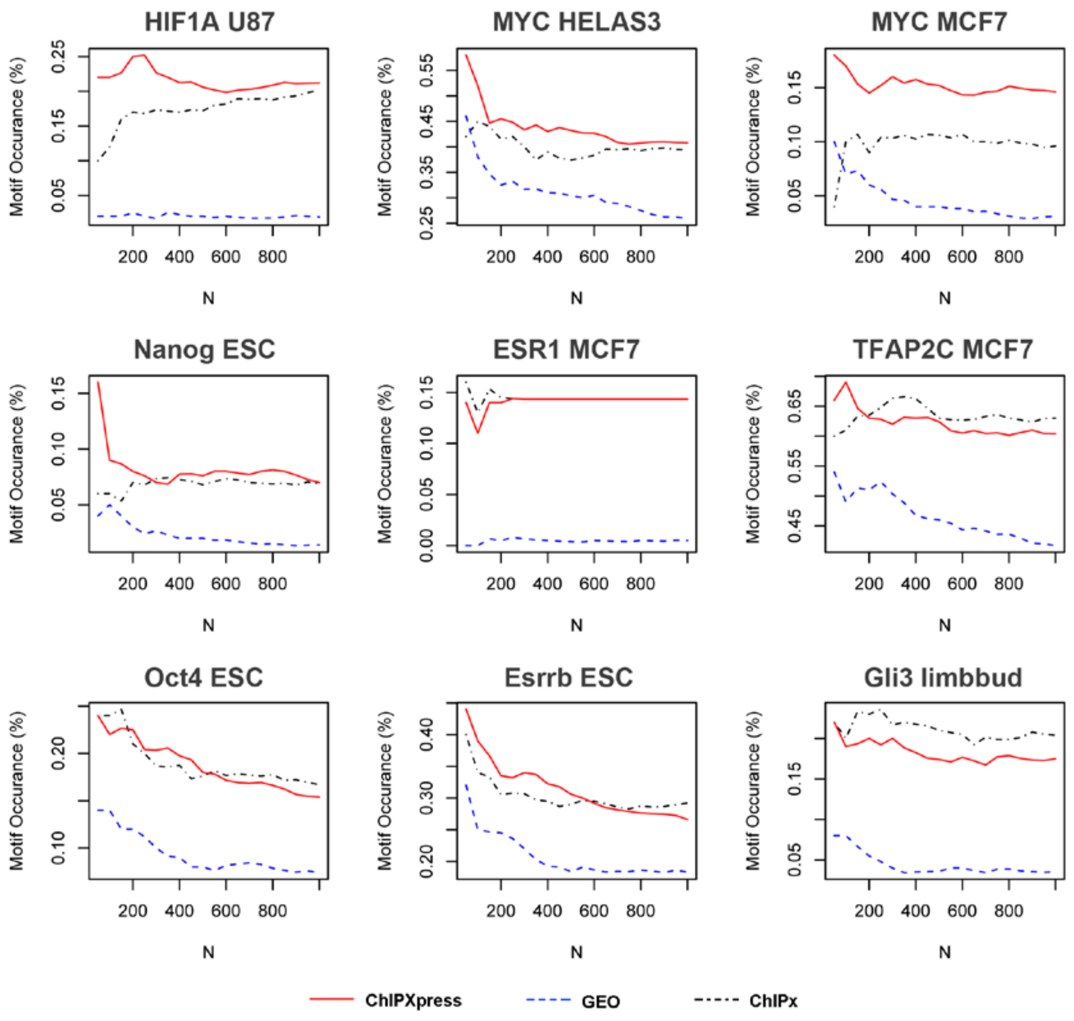

Supplementary Figure 1 – Plot of motif occurrence rate comparing prediction performance of ChIPXpress to ChIPx-only rankings and GEO-only rankings.

Motif occurrence rate among the top N (=50, 100, …, 1000) genes comparing ChIPXpress (solid) rankings to ChIPx-only (dot-dash) and GEO-only (dashed) rankings for *HIF1A* in U87 glioma cells, *MYC* in HelaS3 cervical carcinoma cells, *MYC* in MCF7 breast cancer cells, *Nanog* in embryonic stem cells (ESCs), *ESR1* in MCF7 cells, *TFAP2C* in MCF7 cells, *Oct4* in ESCs, *Esrrb* in ESCs and *Gli3* in limbbud, Overall, ChIPXpress usually improves or maintains the same motif occurrence rate among the top ranked genes, except for *Gli3* in limbbud, where there is a global increase in motif occurrence rate for the ChIPx-only ranking.

Supplemental References

1. Mo Q: **A fully Bayesian hidden Ising model for ChIP-seq data analysis**. *Biostatistics* 2012, **13**:113-128.
2. Ji H, Jiang H, Ma W, Johnson DS, Myers RM, Wong WH: **An integrated software system for analyzing ChIP-chip and ChIP-seq data.** *Nat Biotechnol* 2008, **26**:1293-1300.
3. Li XY, MacArthur S, Bourgon R, Nix D, Pollard DA, Iyer VN, Hechmer A, Simirenko L, Stapleton M, Luengo Hendriks CL, Chu HC, Ogawa N, Inwood W, Sementchenko V, Beaton A, Weiszmann R, Celniker SE, Knowles DW, Gingeras T, Speed TP, Eisen MB, Biggin MD: **Transcription factors bind thousands of active and inactive regions in the Drosophila blastoderm**. *PLoS Biol* 2008, **6**:e27.
4. Farnham PJ: **Insights from genomic profiling of transcription factors**. *Nat Rev Genet* 2009, **10**:605-616.
5. Matys V, Kel-Margoulis OV, Fricke E, Liebich I, Land S, Barre-Dirrie A, Reuter I, Chekmenev D, Krull M, Hornischer K, Voss N, Stegmaier P, Lewicki-Potapov B, Saxel H, Kel AE, Wingender E: **TRANSFAC and its module TRANSCompel: transcriptional gene regulation in eukaryotes**. *Nucleic Acids Res* 2006, **34**:D108-110.
6. Bryne JC, Valen E, Tang MH, Marstrand T, Winther O, da Piedade I, Krogh A, Lenhard B, Sandelin A: **JASPAR, the open access database of transcription factor-binding profiles: new content and tools in the 2008 update**. *Nucleic Acids Res* 2008, **36**:D102-106
